# Identification of a Novel Bat Papillomavirus by Metagenomics

**DOI:** 10.1371/journal.pone.0043986

**Published:** 2012-08-24

**Authors:** Herman Tse, Alan K. L. Tsang, Hoi-Wah Tsoi, Andy S. P. Leung, Chi-Chun Ho, Susanna K. P. Lau, Patrick C. Y. Woo, Kwok-Yung Yuen

**Affiliations:** State Key Laboratory of Emerging Infectious Diseases, Department of Microbiology, The University of Hong Kong, Queen Mary Hospital, Pok Fu Lam, Hong Kong Island, Hong Kong; Centers for Disease Control and Prevention, United States of America

## Abstract

The discovery of novel viruses in animals expands our knowledge of viral diversity and potentially emerging zoonoses. High-throughput sequencing (HTS) technology gives millions or even billions of sequence reads per run, allowing a comprehensive survey of the genetic content within a sample without prior nucleic acid amplification. In this study, we screened 156 rectal swab samples from apparently healthy bats (n = 96), pigs (n = 9), cattles (n = 9), stray dogs (n = 11), stray cats (n = 11) and monkeys (n = 20) using a HTS metagenomics approach. The complete genome of a novel papillomavirus (PV), *Miniopterus schreibersii* papillomavirus type 1 (MscPV1), with L1 of 60% nucleotide identity to Canine papillomavirus (CPV6), was identified in a specimen from a Common Bent-wing Bat (*M. schreibersii*). It is about 7.5kb in length, with a G+C content of 45.8% and a genomic organization similar to that of other PVs. Despite the higher nucleotide identity between the genomes of MscPV1 and CPV6, maximum-likelihood phylogenetic analysis of the L1 gene sequence showed that MscPV1 and *Erethizon dorsatum* papillomavirus (EdPV1) are most closely related. Estimated divergence time of MscPV1 from the EdPV1/MscPV1 common ancestor was approximately 60.2–91.9 millions of years ago, inferred under strict clocks using the L1 and E1 genes. The estimates were limited by the lack of reliable calibration points from co-divergence because of possible host shifts. As the nucleotide sequence of this virus only showed limited similarity with that of related animal PVs, the conventional approach of PCR using consensus primers would be unlikely to have detected the novel virus in the sample. Unlike the first bat papillomavirus RaPV1, MscPV1 was found in an asymptomatic bat with no apparent mucosal or skin lesions whereas RaPV1 was detected in the basosquamous carcinoma of a fruit bat *Rousettus aegyptiacus*. We propose MscPV1 as the first member of the novel Dyolambda-papillomavirus genus.

## Introduction

More than 70% of the emerging infectious disease agents are caused by microbes jumping from animals into human. This has been well exemplified by the highly fatal human infection due to avian influenza A H5N1 in 1997 [1]. The outbreak of severe acute respiratory syndrome (SARS) caused by a novel coronavirus in 2003 [2], confirmed again that microbes can jump species from animals to humans with unpredictable consequence. The human SARS coronavirus was traced to caged civets in the market [3], and later Chinese horseshoe bat, *Rhinolophus sinicus,* was suggested to be a likely reservoir of SARS coronavirus [4]. Bats are ideal incubators for new emerging infectious agents as they are mammals which roosted together and can fly over vast geographical distance [5]. This has reignited the interest in seeking for new bat viruses including many bat coronaviruses and the recent discovery of bat influenza virus [6]. Besides the SARS coronavirus, viruses in bats often infect human through intermediate hosts such as horses for Hendra virus, pigs for Nipah virus, and chimpanzees for Ebola virus [5]. It is therefore important to catalogue as comprehensively as possible the animal viruses present in wild life especially the bats and birds, the food animals such as pigs and cattles, the pet animals such as cats and dogs, and monkeys which are phylogenetically close to humans. Using consensus primer polymerase chain reaction (PCR) screening, we have been able to discover relatively closely related species of virus in many different animals [4], [7]–[23]. However more distant or novel families of virus can only be found by metagnenomics using deep sequencing with the newer generation sequencers [24],[25]. We report in this paper the discovery and characterization of a novel bat papillomavirus (PV) from rectal swab samples randomly collected from asymptomatic wild, food and pet animals using a metagenomic approach.

## Materials and Methods

### Sample collection

This study was performed in strict accordance with local ordinance and the recommendations by the Committee on the Use of Live Animals in Teaching and Research (CULATR) at the University of Hong Kong. The sampling of live animals were approved under permit no. 1048-05 (bats and monkeys) and 2284-10 (stray dogs and cats). All sampling were performed by licensed veterinarians, and anesthesia was given where appropriate; every effort was made to minimize suffering.

Sample collection was carried out in 2006–2007, and was approved by and performed in collaboration with the Department of Agriculture, Fisheries and Conservation (AFCD) of the Hong Kong Special Administrative Region (HKSAR). Collection of animal samples was performed by authorized staff members from AFCD of the HKSAR Government under the supervision of licensed veterinarian from AFCD, HKSAR (http://www.afcd.gov.hk/english/quarantine/qua_awc/qua_awc_leg/qua_awc_leg_dogs/qua_awc_leg_dogs.html).

**Table 1:** Size and position of predicted ORFs and NCR of MscPV1 and the predicted molecular masses of the translated proteins.

ORF	Position	Length		Molecular mass (kDa)	pI
		nt	aa		
E6	1–426	426	141	16.5	9.84
E7	416–691	276	91	9.8	4.99
E1	678–2687	2010	669	75.9	5.62
E2	2626–3768	1143	380	43.4	9.73
E4	3227–3529	303	100	11.5	6.42
L2	3946–5538	1593	530	57.5	6.36
L1	5546–7051	1506	501	56.6	6.84
NCR	7052–7531	480	159	NA	NA

A total of 96 rectal swabs were collected into viral transport medium from 10 types of bats including *Rhinolophus sinicus* (n = 10), *Rhinolophus affinus* (n = 10), *Hipposideros pomona* (n = 16), *Miniopterus pusillus* (n = 10), *Miniopterus schreibersii* (n = 10), *Pipistrellus abramus* (n = 10), *Pipstrellus* spp (n = 9), *Myotis ricketti* (n = 8), *Myotis chinensis* (n = 8), and *Nyctalus noctula* (n = 5). These bats were captured and sampled at 20 different locations in rural areas of the HKSAR, including water tunnels, abandoned mines, sea caves, and forested areas during a 1-year period. Bats were caught by nets during routine conservation procedures by AFCD, HKSAR. Collection of specimens was performed by an authorized veterinarian at the AFCD. Rectal swabs were collected from bats with medium-moistened cotton swab immediately immersed in viral transport medium. These bats were released after sample collection. The samples were collected for a routine surveillance study by AFCD.

The rectal swabs of 9 pigs and 9 cattles were collected and put into viral transport medium in a slaughter house in the New Territories, Hong Kong (Sheung Shui Slaughterhouse), a facility owned and operated by the HKSAR Government. The AFCD is a government department legitimately allowed to perform their duties in collaboration with other departments. The pigs and cattle had been previously slaughtered at the slaughterhouse. Samples from the carcasses were collected for a surveillance study by AFCD staff with departmental authorization.

Rectal swabs of 11 stray dogs, 11 stray cats and 20 monkeys were collected under anaesthesia. The least traumatic techniques were employed for the collection of samples. To minimize sufferings and injury, anaesthesia for restraining were carried out when necessary. The strays dogs and strays cats were kept at AFCD under standard facilities. The stray dogs and cats were euthanized after samplings, as part of the routine procedure of the AFCD. Wild monkeys were caught, temporarily kept in cages for less than one day, sampled and released, also as part of the routine procedure of the AFCD. A licensed AFCD veterinarian was responsible for assessing the well-being of animals for few hours and ensured that they are clinically normal before it was released back to nature. Procedures requiring institutional approval were approved by the Committee on the Use of Live Animals in Teaching and Research (CULATR) at the University of Hong Kong, permit numbers 1048-05 and 2284-10.

**Table 2:** MscPV1 nucleotide and amino acid identities with members of the genera *Kappapapillomavirus, Lambdapapillomavirus, Mupapillomavirus, Nupapillomavirus, Sigmapapillomavirus,* and *Psipapillomavirus.*

Virus^a^	GenBank accession no	Genus	L1		L2		E1		E2	
			nt	aa	nt	aa	nt	aa	nt	aa
CPV6	FJ492744	Lambda	60.0	58.6	45.7	35.4	54.9	44.4	48.0	40.3
CPV1	D55633	Lambda	59.2	57.4	47.8	37.6	53.6	43.2	48.7	41.3
PlpPV1	AY904724	Lambda	59.2	59.5	45.4	37.3	52.7	42.4	47.8	39.4
PlPV1	AY763115	Lambda	59.0	59.1	46.4	36.9	54.5	46.1	51.0	45.3
EdPV1	AY684126	Sigma	58.9	58.3	44.5	32.0	49.9	37.4	45.5	34.6
FdPV1	AF480454	Lambda	58.9	59.1	45.4	37.5	53.3	43.4	48.3	38.9
HPV1	V01116	Mu	58.8	56.5	45.4	35.4	53.4	43.5	48.3	39.2
UuPV1	DQ180494	Lambda	58.8	58.4	45.3	38.2	52.5	42.9	47.1	40.5
SfPV1	K02708	Kappa	58.7	55.9	43.7	31.8	51.5	40.8	44.7	35.6
LrPV1	AY904722	Lambda	58.6	59.0	45.5	37.7	52.6	43.0	50.5	41.4
PcPV1	AY904723	Lambda	58.6	58.4	45.0	36.4	53.5	46.0	47.8	39.2
HPV41	X56147	Nu	58.1	56.4	43.8	34.1	48.3	36.4	44.1	32.3
HPV63	X70828	Mu	57.6	57.9	46.3	36.0	52.3	42.1	50.1	40.7
OcPV1	AF227240	Kappa	57.5	55.6	43.5	32.7	52.2	43.5	48.5	38.3
RaPV1	DQ366842	Psi	52.4	44.8	40.0	27.6	48.7	38.3	45.3	31.7

a CPV6, Canine papillomavirus 6; CPV1, Canine oral papillomavirus; PlpPV1, *Panthera leo persica* papillomavirus type 1; PlPV1, *Procyon lotor* papillomavirus type 1; EdPV1, *Erethizon dorsatum* papillomavirus type 1; FdPV1, *Felis domesticus* papillomavirus type 1; HPV1, Human papillomavirus type 1a; UuPV1, *Uncia uncia* papillomavirus type 1; SfPV1, *Sylvilagus floridanus* papillomavirus type 1; LrPV1, *Lynx rufus* papillomavirus type 1; PcPV1, *Puma concolor* papillomavirus type 1; HPV41, Human papillomavirus type 41; HPV63, Human papillomavirus type 63; OcPV1, *Oryctolagus cuniculus* papillomavirus type 1; RaPV1, *Rousettus aegyptiacus* papillomavirus type 1.

### Sample preparation

The viral transport medium (in which the rectal swab specimens were immersed) were pooled, 100 μl each, and centrifuged at 10000×*g* for 5 min. The supernatant was then filtered through a 0.22 μm filter (Millipore). The filtrates were treated with DNaseI (Roche) and RNaseA (QIAGEN) to remove any extracellular nucleic acids that remained. Total RNA and DNA from the samples were extracted using the QIAamp Viral RNA Mini Kit (QIAGEN) and QIAamp DNA Mini Kit (QIAGEN), respectively. For the total RNA sample obtained, reverse transcription was performed using SuperScript III reverse transcriptase (Invitrogen) and random hexamers (Invitrogen) following the manufacturer's protocol. The cDNA and the previously extracted DNA were separately amplified using the Rapisome pWGA kit (Biohelix).

### 454 sequencing

The amplified DNA was used as a template for GS FLX analysis (Roche/454 Life Sciences) on one-quarter of a PicoTiterPlate according to manufacturer's instruction manual. The purified DNA level was determined by Nanodrop (Thermo Scientific). A total of 120 µg of DNA from the library was run on a 2% agarose gel, yielding a DNA smear. DNA ranging in size from 500 to 1,000 bp was cut from the gel and purified using the QIAquick Gel Extraction Kit (Qiagen). The extremities of the DNA fragments were then polished using T4 polynucleotide kinase. The Roche/454 adaptors were then ligated, and small DNA fragments removed and loaded on the machine according to the manufacturer's protocol (GS FLX Titanium General Library Preparation Kit, Roche).

### Analysis of sequence reads

Sequences were trimmed based on quality score of 99.9% and any sequences less than 30 bp long were deleted. Duplicate sequences were discarded using 454 duplicate clustering workflows (sequence identity threshold of 0.96) based on the CDHIT program [26]. Remaining sequences were compared to a database containing 3,959 complete eukaryotic viral genomes (http://www.ncbi.nlm.nih.gov/genomes/VIRUSES/viruses.html) and the non-redundant protein sequences (nr) database from NCBI (http://www.ncbi.nlm.nih.gov) using tBLASTx and BLASTx, respectively, with an E-value cutoff of 10^−5^ [27]. BLAST results were parsed to save the best hits for each sequence. The best-hit sequences were individually annotated to note the sources of the matching sequences (eukaryotic virus, phage, bacteria and eukaryotes). Sequences were also analyzed using a metagenomic annotation tool, MEGAN version 4.50.6 to assign each sequence into different taxa present in the metagenomic sequences using the NCBI taxonomic database [28]. All unmapped reads were *de novo* assembled separately using MIRA to identify previously undetected virus [29].

### *De novo* metagenomic assembly

*De novo* assembly of the metagenome was performed using MIRA to confirm isolation of viral genomes using an assembly option with minimum read length of 80 and base default quality of 10 [29]. There were 1,283 contigs ranging in size from 91 to 3,722 bp for human samples, and 1,960 contigs from 116 to 11,170 bp for animal samples. Contigs were compared to the database containing 3,959 complete eukaryotic viral genomes and the nr database from NCBI using tBLASTx and BLASTx, respectively, with an E-value cutoff of 10^−5^ to assign taxonomy.

### Confirmation of the presence and completion of the assembled genome in the original specimen

After the incomplete genome of this novel virus was assembled from the metagenomic dataset, specific primers were designed to fill the gaps for the completion of this viral genome (primers available on request). For the confirmation of the host specimen containing this novel virus, DNA of each original specimen was subjected to PCR by using the primers specific for the L1 gene amplifying a 444 bp fragment between positions 483 to 926 (forward primer LPW11859 5′-GGCTCTCGGTGAGCACT-3′ and reverse primer LPW11861 5′-CAGTAAGGTCTGTTGAACAGTT-3′). The PCR mixture consisted of DNA template, PCR Buffer II at 1× (Applied Biosystems), 2 mM MgCl_2_, 200 μM of each dNTPs and 0.625 U Ampli*Taq* Gold DNA polymerase (Applied Biosystems). The mixtures were amplified in thermal cycler 9700 (Applied Biosystems), with a hot start of 95°C for 5 min, followed by 40 cycles of 95°C for 1 min, 55°C for 1 min and 72°C for 1 min and a final extension at 72°C for 10 min. PCR product was gel-purified using the QIAquick gel extraction kit (Qiagen). Both strands of the PCR products were sequenced with an ABI Prism 3700*xl* Genetic Analyser (Applied Biosystems) by using the PCR primers.

### Distance measurements and phylogenetic analysis

The nucleotide global multiple sequence alignments were constructed for different open reading frames (ORFs) with 214 PVs based on the corresponding amino acid alignment using MUSCLE v3.7 [30] implemented in Seaview v4.1 as described previously [31], [32]. The pairwise identity values from nucleotides and proteins were calculated using MEGA5 [33]. Only the PV core *early (E) ORFs E1* and *E2* and the *late (L) ORFs L1* and *L2* were included as only these ORFs are ubiquitous present in all characterized PVs. L1 nucleotide sequences of MscPV1 and 78 PVs with complete genomes, representing all presently classified genera, were used for phylogenetic analysis. Maximum likelihood trees were constructed using PhyML with GTR+I+G model [34]. Modelgenerator was used to obtain the model for the likelihood analysis [35].

### Genome analysis

Putative ORFs were predicted using ORF Finder and then searched for similarities with other proteins using BLASTP. Theoretical isoelectric points and molecular masses were estimated using Compute pI/Mw (http://web.expasy.org/compute_pi/). Proteins were analyzed for unique domains with InterProScan [36].

### Prevalence of MscPV1 in bats

Prevalence of MscPV1 in *M. schreibersii* was further investigated by PCR screening of 419 additional samples (mouth swabs [n = 210], rectal swab [n = 127], anal swabs [n = 2], and urine samples [n = 80]) obtained from 257 bats using primers specific for the L1 gene amplifying a 444 bp fragment between positions 483 to 926 (forward primer LPW11859 5′-GGCTCTCGGTGAGCACT-3′ and reverse primer LPW11861 5′-CAGTAAGGTCTGTTGAACAGTT-3′).

### Nucleotide sequence accession number

The nucleotide sequence of the genome of MscPV1 has been lodged within the GenBank sequence database under accession no. JQ692938.

## Results

### Identification of a novel papillomavirus

**Figure 1:**
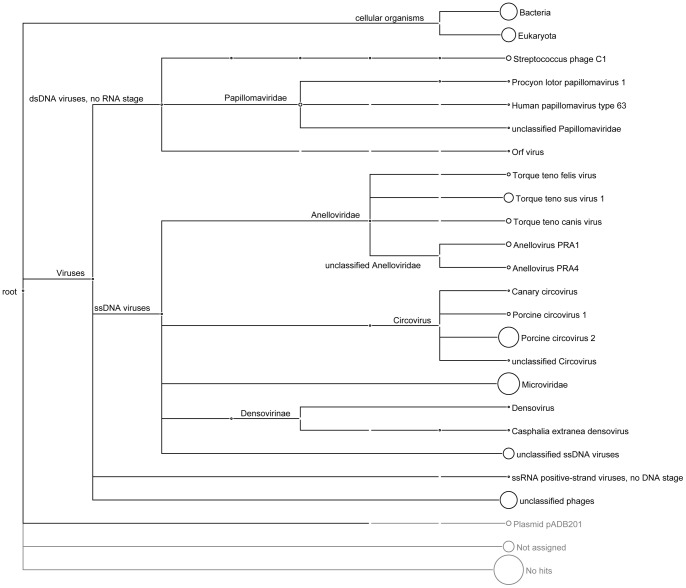
MEGAN tree with taxonomic assignments. The distribution of the sequence reads through blastx analysis against the nr database. Size of circles located next to taxa are proportional to the total number of reads identified. Not assigned contains those reads that are not assigned by the least common ancestor algorithm. No hits contains those reads that did not return any significant alignments to the nr database.

Approximately 3% of the sequence reads generated from these animal samples were assigned to eukaryotic viral sequences by the BLAST nr protein database. The majority of the viral-like sequences were similar to single-stranded, negative-sense, circular DNA viruses, with the largest proportion of the sequences showing homology to porcine circovirus. The next large group of the sequences matched to another member of the *Circoviridae* family, torque teno virus, including torque teno felis virus, torque teno sus virus 1 and torque teno canis virus. The remaining viral-like sequences shared homology to canary circovirus, anellovirus and densovirus in which densovirus is a linear single-stranded DNA virus. In addition, many sequences were categorized as phage-related genes (Fig. 1). The majority of these sequences were related to porcine circovirus in animal samples. Twenty two sequence reads and one contig in animal sample were related to PVs with amino acid identity ranged from 42% to 73%. These hits cover about 70% of the viral genome, which we named *Miniopterus schreibersii* papillomavirus type 1 (MscPV1), since the sample was isolated from a Common Bent-wing Bat (*M. schreibersii*). This bat is a female adult bat collected on 29 December 2006 in Tung Tsz, Hong Kong. By connecting gaps between sequenced viral fragments based on PV sequences, the complete genome of the novel PV was acquired.

### Characterization of MscPV1 complete genome

**Figure 2:**
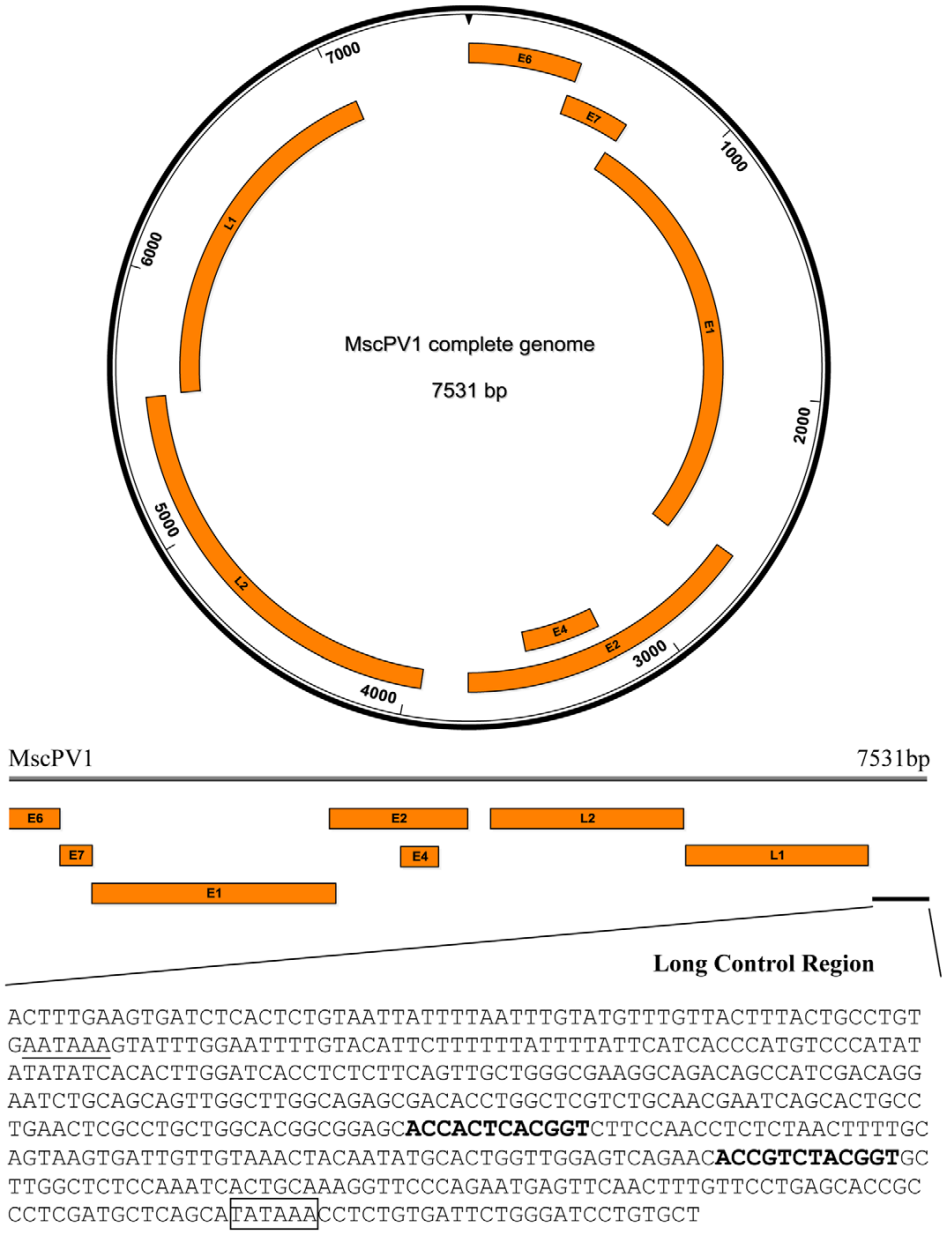
Circular and linear genome maps of *Miniopterus schreibersii* papillomavirus type 1 (MscPV1). Characteristic features of the long control region of MscPV1, showing genomic locations of E2 binding sites (bold), polyadenylation sites (underlined), and TATA box (boxed).

The complete genome of MscPV1 was 7,531 bp in length with a G+C content of 45.8%. The MscPV1 genome contains the typical PV ORFs, coding for five putative early proteins (E6, E7, E1, E2, E4), and two putative late capsid proteins (L2 and L1) (Fig. 2 and Table 1).

The MscPV1 E6 contains two conserved zinc binding domains (CXXCX_29_CXXC), separated by 36 amino acids, whereas the MscPV1 E7 contained one slightly modified domain (CXXCX_30_CXXC), but no retinoblastoma tumour suppressor (pRB)-binding domain (LXCXE) [37]. The E1 ORF codes for the largest MscPV1 protein (669 aa), and contains the conserved ATP-binding site of the ATP-dependent helicase (GXXXXGK(T/S)) [38]. This sequence is GPPDTGKS in MscPV1. The E2 protein has the typical C-terminal DNA-binding domain and the N-terminal transactivation domain [39], [40]. The MscPV1 E4 gene is located in the E region and overlaps with E2 but is transcribed in a different reading frame. An LLXLL motif is found at the N-terminus of viral E4 [41]. Downstream from the leucine-rich region is a proline-rich region. PV E4 proteins usually have high proline content (15–20% on average), MscPV1 E4 protein also has the typical high proline content (15 proline residues out of 106 aa).

Both L1 and L2 contain a series of arginine and lysine residues at their carboxy termini, likely to function as a nuclear localization signal. The long control region (NCR) usually contains several regulators of the PV replication. In MscPV1, the NCR is 481 bp and demonstrates an E1-binding site (TGATTGTTGTAAACTAC) flanked by two typical palindromic E2-binding sites (ACCN_6_GGT) [42]. At its 5′ end, the NCR also contains one polyadenylation site (AATAAA) which is necessary for the processing of the L1 and L2 capsid mRNA transcript [43]. In the 3′ end, the MscPV1 NCR contains a classical TATA box (TATAAA) of the E6 promotor, located 26 nucleotides upstream of the E6 start codon (Fig. 2).

### Phylogenetic analysis and sequence similarity to other papillomaviruses

**Figure 3:**
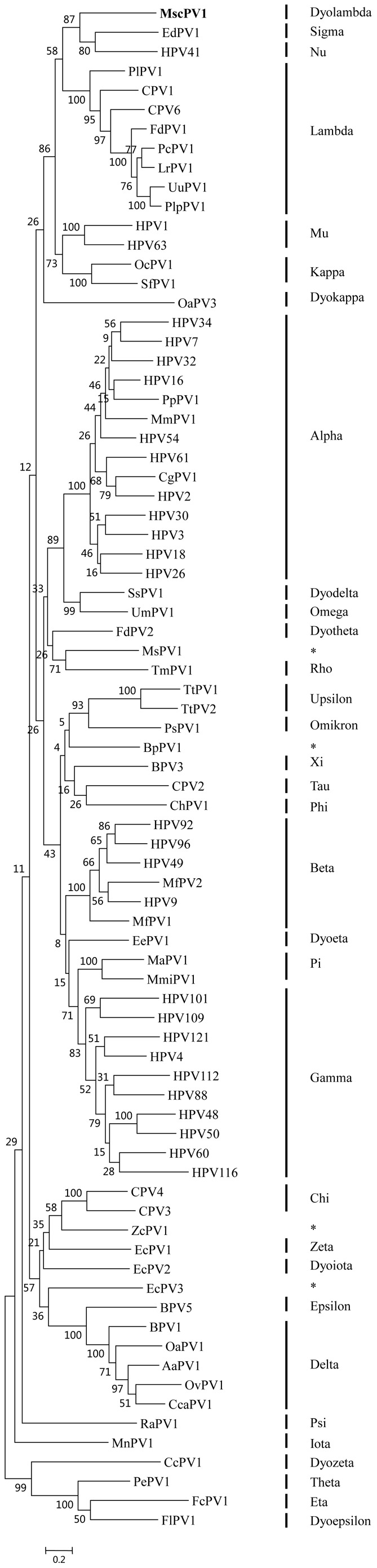
Maximum likelihood phylogenetic tree of the L1 nucleotide sequences of 79 PVs. The PV genus of each strain is indicated. PVs with putative PV genera that are currently unclassified are marked by asterisks. The PV discovered in this study is shown in bold. Scale bar indicates 0.2 inferred substitutions per site. AaPV, *Alces alces* papillomavirus; BpPV, *Bettongia penicillata* papillomavirus; BPV, Bovine papillomavirus; CcaPV, *Capreolus capreolus* papillomavirus; CcPV, *Caretta caretta* papillomavirus; CgPV, *Colobus guereza* papillomavirus; ChPV, *Capra hircus* papillomavirus; CPV, Canine papillomavirus; EcPV, *Equus caballus* papillomavirus; EdPV, *Erethizon dorsatum* papillomavirus; EePV, *Erinaceus europaeus* papillomavirus; FcPV, *Fringilla coelebs* papillomavirus; FdPV, *Felis domesticus* papillomavirus; FlPV, *Francolinus leucoscepus* papillomavirus; HPV, Human papillomavirus; LrPV, *Lynx rufus* papillomavirus; MaPV, *Mesocricetus auratus* papillomavirus; MfPV, *Macaca fascicularis* papillomavirus; MmiPV, *Micromys minutus* papillomavirus; MmPV, *Macaca mulatta* papillomavirus; MnPV, *Mastomys natalensis* papillomavirus; MscPV, *Miniopterus schreibersii* papillomavirus; MsPV, *Morelia spilota spilota* papillomavirus; OaPV, *Ovis aries* papillomavirus; OcPV, *Oryctolagus cuniculus* papillomavirus; OvPV, *Odocoileus virginianus* papillomavirus; PcPV, *Puma concolor* papillomavirus; PePV, *Psittacus erithacus timneh* papillomavirus; PlpPV, *Panthera leo persica* papillomavirus; PlPV, *Procyon lotor* papillomavirus; PpPV, *Pygmy chimpanzee* papillomavirus; PsPV, *Phocoena spinipinnis* papillomavirus; RaPV, *Rousettus aegyptiacus* papillomavirus; SfPV, *Sylvilagus floridanus* papillomavirus; SsPV, Sus scrofa papillomavirus; TmPV, *Trichechus manatus latirostris* papillomavirus; TtPV, *Tursiops truncatus* papillomavirus; UmPV, *Ursus maritimus* papillomavirus; UuPV, *Uncia uncia* papillomavirus; ZcPV, *Zalophus californianus* papillomavirus.

Phylogenetic analysis confirmed that MscPV1 forms a genetic lineage that is distinct from the previously reported PVs with complete genome (Fig. 3). Comparison of L1 gene showed that MscPV1 had 60% nucleotide and 58.6% amino acid identity to the closest related PV, Canine papillomavirus 6 (Table 2). MscPV1 also shared only 52.4% nucleotide identity to another PV isolated from an Egyptian fruit bat (*Rousettus aegyptiacus*) (Table 2) [44]. MscPV1 cannot be placed in one of the existing genera, it therefore represents the first member of a novel PV genus, Dyolambda-papillomavirus, according to the classification criteria [31], [45].

### Prevalence of MscPV1 in bats

None of the 419 samples from 257 *M. Schreibersii* bats screened by PCR was positive.

## Discussion

Virus discovery has traditionally been done by phenotypic techniques such as animal inoculation or chick embryo inoculation which are later replaced by tissue culture. With major advance in molecular and sequencing technology, many viruses that may not adapt to grow in tissue cultures were discovered by PCR and sequencing in various formats such as consensus primer PCR with or without hybridization on microarray, rolling circle amplification for virus with circular genome, representational difference analysis by subtractive hybridization, sequence independent PCR amplification with shotgun sequencing. The advent of high-throughput sequencing has allowed the discovery of many novel animal viruses such as novel species of porcine circoviruses, astroviruses and bocaviruses [46], [47], novel sapoviruses, noroviruses, dependoviruses in sealions [48], novel kobuvirus and sapovirus in diarrheal dogs [49], novel hepacivirus in dogs affected by outbreak of respiratory illness [50], novel anellovirus in sea seals [51], novel astrovirus in brain tissue of mink suffering from shaking mink syndrome [52] and many other virus families in human [53], turkey [54], bat guano [55], rodent excreta [56] and insects [57], [58]. Metagenomics has also led to the study of the viral diversity and community in hosts and the association of virus and disease [59]–[61].

In this study, we report the second bat PV, MscPV1, with only 52.4% nucleotide identity to the Egyptian fruit bat (*Rousettus aegyptiacus*) papillomavirus RaPV1. Compared to other PVs, the highest nucleotide and amino acid identities, from CPV6, were only 60% and 58.6%. According to the published classification criteria, MscPV1 should be designated the first member of a novel PV genus, Dyolambda-papillomavirus [31], [45].

Comparing MscPV1 with the phylogenetically closely-related PVs, namely, RaPV1, HPV41, EdPV1 and CPV6, all of MscPV1, RaPV1, EdPV1 and CPV4 contain the typical PV ORFs, coding for five putative early proteins (E6, E7, E1, E2, E4), and two putative late capsid proteins (L2 and L1). The genome of HPV41 consists of an additional E5 ORF located between E4 and L2 ORFs and three additional short ORFs, X, Y and Z downstream of L1 [62]. The E5 ORF, which is absent in MscPV1, exists in genital HPVs and in the BPV-1 related fibropaillomaviruses, codes for the E5 protein, which is associated with transformation of host cells and carcinogenesis [62], [63]. The predicted E7 protein of MscPV1 contains a modified zinc-binding domain with 30 amino acids (X_30_) between the two instances of CXXC. This nonclassical motif was also identified in HPV41 and RaPV1 as well as BPV6, CPV2, CPV7, CcaPV1, HPV4, HPV65, HPV95 and HPV116. The E7 of EdPV1 exhibits the classical CXXCX_29_CXXC motif, whereas CPV6 has the X_28_ modified motif. The E1 of MscPV1 contains the conserved ATP-binding site of the ATP-dependent helicase (GPPDTGKS), which is identical to that of RaPV1; in comparison, the motif is GPPNTGKS in EdPV1 and CPV6, and GPSDTGKS in HPV41. This sequence conservation is not unexpected, given the drastic decrease of ATPase activity upon mutation of just the first proline or the lysine residue of the motif demonstrated by a mechanistic study [64]. In the NCR of both MscPV1 and RaPV1, two copies of the 12-basepair E2 protein-binding motif ACCN_6_GGT are found. The genomes of HPV41 and EdPV1, notably, do not contain this consensus nucleotide sequence; their E2 binding sites are represented by the sequences ACCN_6_GTT, AACN_6_GGT, and AACN_6_GTT [62], [65].

Family *Papillomaviridae* is a large family of small, non-enveloped, double stranded DNA viruses which infect cutaneous and mucosal epithelium. Given the association between PV and cancers in humans and other animals, it is not surprising that the first bat PV was found in the basosquamous carcinoma of a fruit bat. PVs are stable and slow-evolving viruses, with an estimated mutation rate of 0.73 to 1.2×10^−8^ nucleotide substitutions per base per year [66], [67]. No genomic recombination has ever been documented. Novel PVs, therefore, have been believed to descend from the slow accumulation of point mutations and different ancient PV lineages have possibly co-evolved and co-speciated with their vertebrate host species [68], [69]. Nonetheless, a more recent study showed that recombination of PV contributes significantly to the evolution of PV, and the role of host transfer cannot be neglected. Notably, both were observed in the HPV41-EdPV1 clade [70]. This is concordant with the current study, in which nucleotide and amino acid sequence analysis demonstrated a higher degree of similarity between our novel MscPV1 and EdPV1 in North American porcupine, instead of another bat PV, RaPV1. We note, however, our findings may only represent an additional exception and do not refute the generalization that PVs evolve mainly by co-evolution with hosts instead of interspecies transmission, recombination or other horizontal genetic transfer events. While current evidence suggests that host shift may have contributed to the emergence of this lineage of bat PV, as evidenced by the different clades in which the MscPV1 and we reckon that there may be bat PV lineages that are yet to be discovered, and such “missing-links” may enable the evolutionary history of MscPV1 to be put into context, i.e. whether the MscPV1 has undergone unexpected, rapid divergent evolution or indeed represents a lineage of bat PVs that has arisen from host shift. Although divergence time estimation using the L1 nucleotide sequence of MscPV1 demonstrated a later divergence compared to the host species (Fig. S1), it differed from the estimate based on E1 nucleotide sequences (Fig. S2) and does not in itself substantiate the host transfer (Supporting Information S1). In the current study, the more convincing evidence seemingly still resides in the sister taxa status of MscPV1 and HPV41/EdPV1. The relative importance of virus-host co-divergence and interspecies transmission in driving the genomic evolution of PV remains to be debated [71].

Unlike the first report of bat PV, no obvious skin or mucosal lesion was noted by the attending veterinarian and the bat was probably latently infected with PV. *M. schreibersii* is a cave-dwelling bat with body weight ranged from 11 to 18 g. It roosts in abandoned mines, water tunnels, drainage and weep holes of the water catchment. According to the baseline surveys of the AFCD of Hong Kong, *M. schreibersii* is considered as common and widespread throughout Hong Kong countryside with a colony size from 50 individuals to several hundreds, often associated with *M. pusillus*, *Myotis pilosus* and *M. chinensis* in their roosting sites in summer. In overseas studies, it is reported as a migratory bat species which may travel a fairly long distance in spring to find their breeding sites [72]. Given the possibilities of asymptomatic carriage and long-distance, interspecies transmission, further studies are warranted to elucidate the evolutionary origin and epidemiology of this newly proposed genus of bat PV.

## Supporting Information

Figure S1 **Estimation of the time to the most recent common ancestor for MscPV1 using L1.** The maximum likelihood tree constructed by PhyML using L1 were used to estimate the divergence times in MEGA5. Virus name abbreviations are the same as those in the Fig. 3 legend. MscPV1 was bolded.
(TIF)

Figure S2 **Estimation of the time to the most recent common ancestor for MscPV1 using E1.** The maximum likelihood tree constructed by PhyML using E1 were used to estimate the divergence times in MEGA5. Virus name abbreviations are the same as those in the Fig. 3 legend. MscPV1 was bolded.
(TIF)

Supporting Information S1 (DOC)
