# Supplementary figures and images for: Identification of a Novel Bat Papillomavirus by Metagenomics
Source: PLoS One. 2012 Aug 24;7(8):e43986. doi: 10.1371/journal.pone.0043986 (PMC3427170; doi:10.1371/journal.pone.0043986)

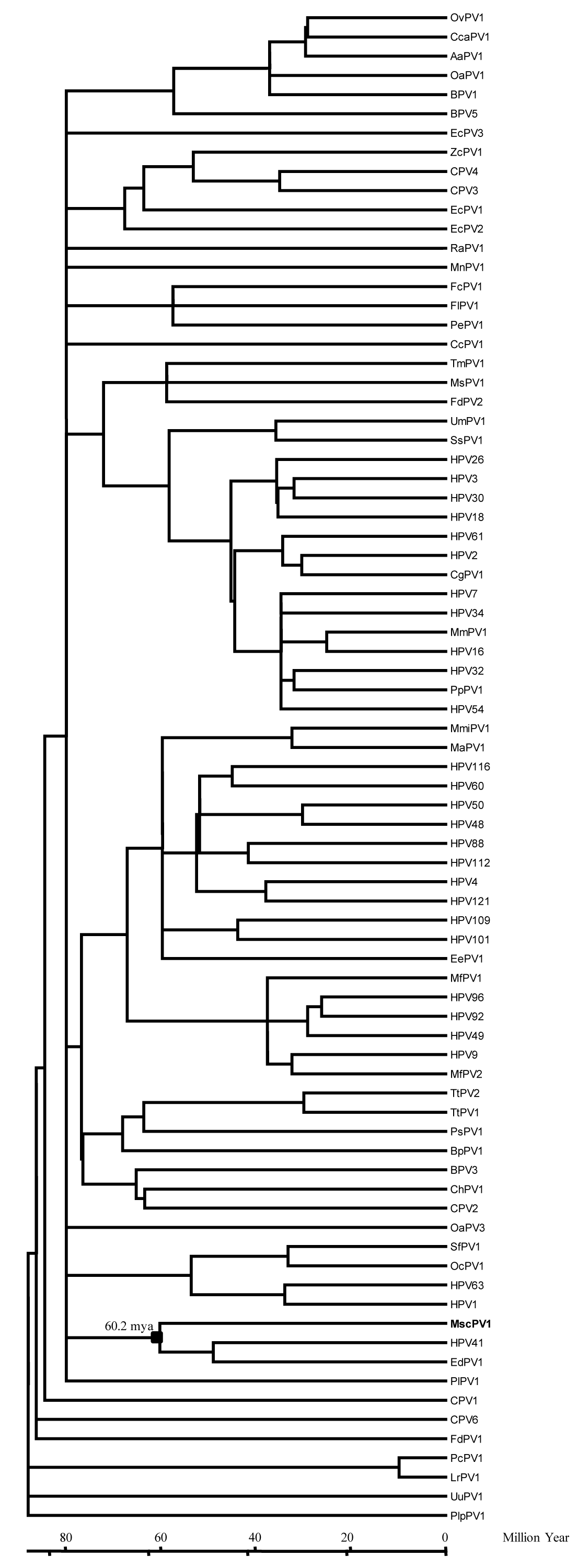

Supplement: Figure S1 — Estimation of the time to the most recent common ancestor for MscPV1 using L1. The maximum likelihood tree constructed by PhyML using L1 were used to estimate the divergence times in MEGA5. Virus name abbreviations are the same as those in the Fig. 3 legend. MscPV1 was bolded. (TIF) [file pone.0043986.s001.tif]

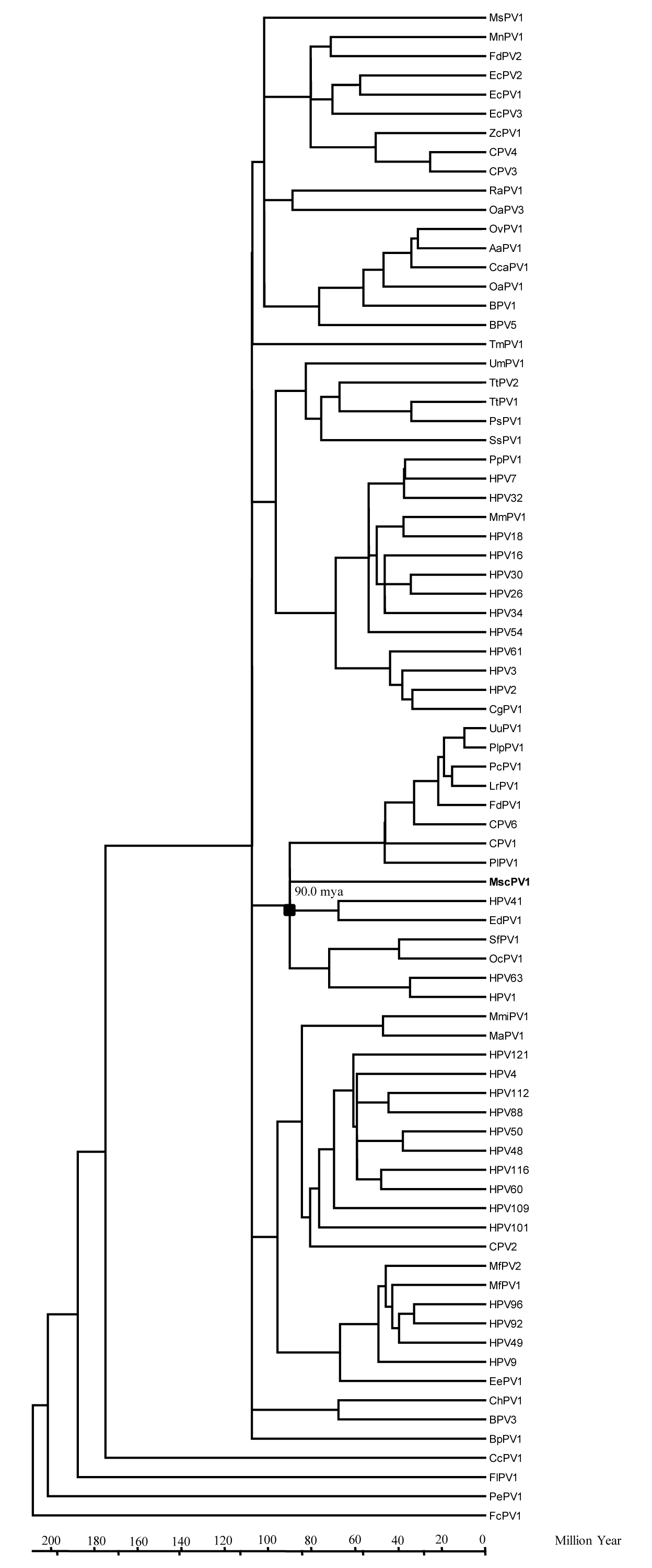

Supplement: Figure S2 — Estimation of the time to the most recent common ancestor for MscPV1 using E1. The maximum likelihood tree constructed by PhyML using E1 were used to estimate the divergence times in MEGA5. Virus name abbreviations are the same as those in the Fig. 3 legend. MscPV1 was bolded. (TIF) [file pone.0043986.s002.tif]
