# Supplementary material for: Identification of a Novel Bat Papillomavirus by Metagenomics
Source: PLoS One. 2012 Aug 24;7(8):e43986. doi: 10.1371/journal.pone.0043986 (PMC3427170; doi:10.1371/journal.pone.0043986)
Supplement: Supporting Information S1 — (DOC) [file pone.0043986.s003.doc]

**Supporting Information S1**

### Identification of a novel bat papillomavirus by metagenomics

### Herman Tse^#^, Alan K. L. Tsang^#^, Hoi-Wah Tsoi^#^, Andy S. P. Leung, Chi-Chun Ho, Susanna K. P. Lau, Patrick C. Y. Woo, Kwok-Yung Yuen*

### From the State Key Laboratory of Emerging Infectious Diseases, Department of Microbiology, The University of Hong Kong, University Pathology Building, Queen Mary Hospital, Hong Kong.

**MATERIALS AND METHODS**

**Estimated time of divergence**

Estimate of divergence time was calculated using MEGA 5 . The clock rates of 9.6 × 10^-9^ substitutions per site per year (S.D. = 2.1 × 10^-10^ substitutions per site per year) for the L1 gene, and 7.1 × 10^-9^ substitutions per site per year (S.D. = 1.5 × 10^-10^ substitutions per site per year) for the E1 gene, were obtained from the mean substitution rates estimated by Shah *et al.* . As host divergence events unlikely serve as definitive calibration points globally in the tree in view of the possible host switch, this estimation only adapted the substitution rates and assumed a strict clock, under which the L1 and E1 genes of the PV lineage in the current study did not undergo significantly accelerated evolution upon adaptation to the new host, and is therefore likely biased towards an older divergence time.

**RESULTS**

**Estimated time of divergence**

MscPV1 and the HPV41/EdPV1 lineage were estimated to have diverged from a common ancestor 60.2 million years ago (Mya) (58.9 – 61.6 Mya) using L1 sequences (Fig. S1). When compared to the time of divergence estimated for the hosts, 94.4 Mya for the split between *Miniopterus schreibersii* and *Homo sapiens* / *Erethizon dorsatum* [3], the estimated time of viral divergence was found to be later than that of the host species. The divergence time estimated using the E1 gene was older, at 90.0 Mya (88.1 – 91.9 Mya), although the polytomy in the linearised tree suggested that the divergence of MscPV1 from its sister taxa could not be adequately resolved by E1 gene sequences using this method (Fig. S2).

**REFERENCES**

1. Tamura K, Peterson D, Peterson N, Stecher G, Nei M, et al. (2011) MEGA5: molecular evolutionary genetics analysis using maximum likelihood, evolutionary distance, and maximum parsimony methods. Mol Biol Evol 28: 2731-2739.

2. Shah SD, Doorbar J, Goldstein RA (2010) Analysis of host-parasite incongruence in papillomavirus evolution using importance sampling. Mol Biol Evol 27: 1301-1314.

3. Hedges SB, Dudley J, Kumar S (2006) TimeTree: a public knowledge-base of divergence times among organisms. Bioinformatics 22: 2971-2972.
